# A Neonate with FNAIT Supported by Placental Chronic Histiocytic Intervillositis and Confounded by Maternal Preeclampsia: A Case Report

**DOI:** 10.1055/a-2703-4068

**Published:** 2025-10-01

**Authors:** Hannah White, Amelia Sybenga

**Affiliations:** 1 Department of Medical, Larner College of Medicine at the University of Vermont, Burlington, Vermont; 2 Department of Surgical/Perinatal Pathology, University of Vermont Medical Center, Burlington, Vermont

**Keywords:** FNAIT, CHI, anti-HPA antibody

## Abstract

**Introduction:**

Fetal and neonatal alloimmune thrombocytopenia (FNAIT) occurs in the setting of maternal anti-human platelet antigen (anti-HPA) antibodies against paternally derived fetal platelet antigens. Recent studies have also demonstrated an association between chronic placental inflammation and FNAIT, specifically low-grade chronic histiocytic intervillositis (CHI). We present a neonate with profound thrombocytopenia after delivery with co-occurring CHI, whose platelet counts recovered rapidly with platelet transfusions, born to a primigravida mother with late-onset preeclampsia.

**Case Report:**

A male neonate was born at 40 weeks to a mother who had no known history of pregnancies, miscarriages, or transfusions. The mother developed severe preeclampsia during the induction of labor. Shortly after delivery, a physical exam of the infant showed inappropriate bruising on the heels of both feet, scattered petechiae on the hard palate, a hematoma on the left thigh after a vitamin K shot, and a bruise on the upper left abdomen. His platelet count was found to be 7,000. Platelet count rose to 94K by day 3 of life following transfusions. Placental pathology confirmed CHI. Maternal testing revealed anti-HPA-1 antibodies supporting FNAIT.

**Conclusion:**

This case highlights a potential relationship between maternal alloimmune response and preeclampsia. It also highlights the importance of considering FNAIT as a diagnosis in a neonate presenting with thrombocytopenia regardless of maternal preeclampsia, and the importance of submitting the placenta for a pathology exam.

Fetal and neonatal alloimmune thrombocytopenia (FNAIT) occurs in the setting of maternal anti-human platelet antigen (anti-HPA) antibodies against paternally derived fetal platelet antigens. 1 The most common antibody is anti-HPA-1a, although others have been implicated. These antibodies develop following exposure to HPA antigens absent in the mother. Maternal immunoglobulin G (IgG) antibodies form against fetal platelet antigens and are transported across the placenta, causing destruction of fetal platelets and leading to thrombocytopenia and bleeding in the neonate. 2 FNAIT has been reported in as high as 1/1,000 live births and likely remains underdiagnosed, as antenatal screening for FNAIT is not routinely performed. 3

Other causes of neonatal thrombocytopenia include prematurity, maternal pregnancy-induced hypertensive disorders, and antenatal infection. Rarer etiologies include immune mediated destruction, such as maternal autoimmune disease (lupus) or drug induced/dependent antibodies; chromosomal abnormalities and other genetic disorders; increased clearance of platelets as seen in fetal hypersplenism, Kaposiform hemangioendothelioma, and type 2B von Willebrand disease; bone marrow or specific lineage failure; and marrow replacing neoplasms, such as congenital leukemias or bone marrow involvement by neuroblastoma. 4

Recent studies have also demonstrated an association between chronic placental inflammation and FNAIT, specifically low-grade chronic histiocytic intervillositis (CHI). 3 Furthermore, it is reported that more than 40% of mothers with anti-HPA-1a antibodies have placentas with CHI, which may contribute to the fetal growth restriction and reduced birth weight associated with FNAIT. CHI is theorized to represent an allogeneic humoral (antibody-associated) rejection following the transfer of IgG antibodies from mother to fetus. Both CHI and FNAIT often recur in subsequent pregnancies, although severity is unpredictable.

We present a neonate with profound thrombocytopenia after delivery with co-occurring CHI, whose platelet counts recovered rapidly with platelet transfusions, but without IVIG therapy, born to a primigravida mother with late-onset preeclampsia.

## Case Report

A male neonate was born at 40 weeks' gestational age to a mother who had no known history of pregnancies, miscarriages, or transfusions. The pregnancy was complicated by O and Rh− blood type, obesity (BMI = 53), and late-onset preeclampsia with severe features, diagnosed by severe range blood pressures (SRBPs) and elevated urine protein-to-creatinine ratio (0.4), during induction of labor, requiring magnesium administration. For SRBPs, she received hydralazine 10 mg twice and labetalol 20 mg thrice, and ultimately underwent cesarean section for arrest of descent. Birth weight was 3,505 g, appropriate for age, 5 and APGAR scores were 9 and 9, respectively, but, shortly after delivery, physical exam showed inappropriate bruising on the heels of both feet, scattered petechiae on the hard palate, a hematoma on the left thigh where his Vitamin K shot was administered, and a 1 cm × 1 cm bruise on the upper left abdomen. He was transferred to our medical center for signs of thrombocytopenia.

**Fig. 1:**
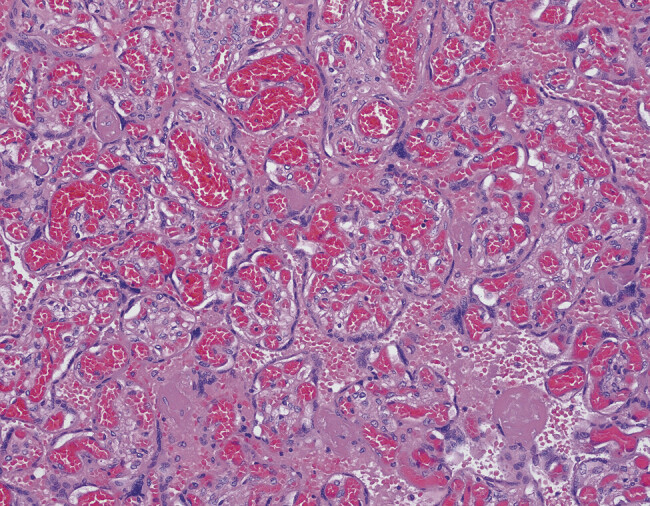
Chorionic villi with intravillous stromal hemorrhages and increased circulating nucleated red blood cells (H&E, 10 × ).

**Fig. 2:**
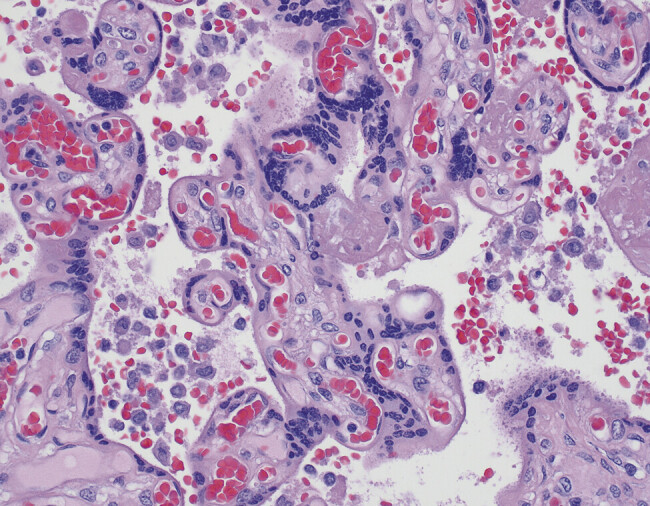
Aggregates of intervillous histiocytes (H&E, 40 × ).

**Fig. 3:**
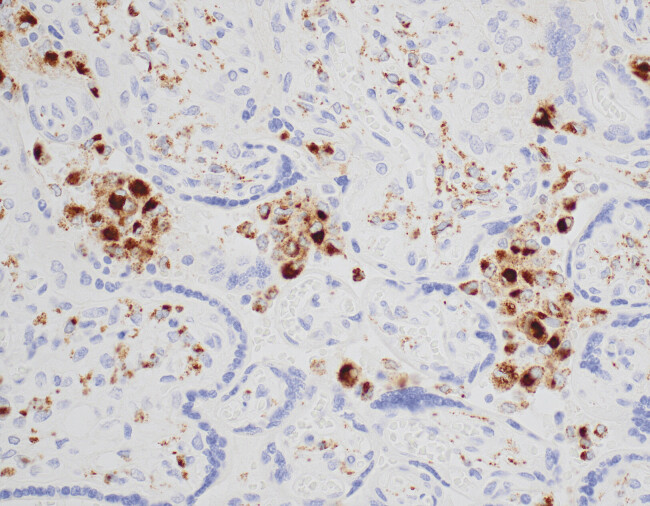
Intervillous histiocytes highlighted by CD68 immunostain (40×).

Labs from cord blood at the referring hospital revealed a platelet count of 10,000 and a subsequent serum platelet count of 9,000. On admission to our unit, his serum platelet count was 7,000 with an immature platelet fraction of 32%. His blood type was A and Rh − . He was transfused with platelets from a random donor at 10 mL/kg, but his platelet count rose insufficiently from 7K to 29K, and he was given a second platelet transfusion the following day, this time from an HPA-1a negative donor, at 15 mL/kg. His platelet count rose to 94K by day 3 of life, with resolution of clinical symptoms of thrombocytopenia. Abdominal ultrasound and head ultrasound were unremarkable. At this time, the differential diagnosis for the thrombocytopenia was preeclampsia versus neonatal alloimmune thrombocytopenia (NAIT); however, preeclampsia was initially favored. Placental pathology was also performed. The placenta was 655 g (>90th percentile for 40 weeks of gestational age). Histology showed fetal normoblastemia without erythroblastosis and prominent villous stromal hemorrhages ( Fig. 1 ). In addition, patchy aggregates of intervillous histiocytes were noted throughout the intervillous space ( Fig. 2 ), confirmed with CD68 immunostain, suspicious for low-grade CHI ( Fig. 3 ).

Subsequent send-out laboratory results confirmed paternal HPA-1a positive platelets, maternal HPA-1a negative platelets, and a maternal HPA-1a antibody, supporting the diagnosis of NAIT. Due to the rise in his platelet count and resolution of clinical symptoms, the mother and baby were discharged home on day 3 to be followed by hematology and his pediatrician. Following discharge, the patient had no new symptoms or signs of thrombocytopenia, and his platelet counts were 218K and 290K at 13 days and at 3 weeks of life, respectively.

## Discussion

HPA-associated alloimmunization in first pregnancies is not uncommon. 6 7 One study showed 63% of severe FNAIT cases occurred in first-time pregnancies, with worsening outcomes when clinical features of FNAIT appeared at an earlier gestational age. 6 The mechanism of alloimmunization is not well understood; however, HPA-1a antigens are also expressed on placental trophoblast cells, a possible source of alloimmunization. 8

The CHI in this placenta is mild and similar to prior reports of CHI in the placenta of FNAIT cases. 3 Interestingly, CHI has also been loosely associated with maternal hypertensive disorders, 9 and preeclampsia has been reported complicating FNAIT, raising the question about preeclampsia as a symptom of FNAIT in this case, rather than an independent disease. 10

The extravillous trophoblast (EVT) plays a critical role in maternal vascular remodeling and regulation of placental blood flow. EVT dysfunction is thought to be the primary cause of preeclampsia. Inappropriate EVT function leads to reduced blood flow and placental hypoxia, leading to a hypoxia-induced inflammatory response and the clinical features of preeclampsia. HPA-1a antigens are also expressed on EVT cells. It is interesting to consider that the development of maternal HPA-1a antibodies during this pregnancy may have led to an alloimmune response against EVT cells, leading to dysregulated placental blood flow and the development of late-onset preeclampsia. 11

## Conclusion

This case is unique as it highlights a relationship between maternal alloimmunity and placental dysfunction and provides further support for a potential causal relationship between failed maternal immune tolerance and the development of preeclampsia.
